# Neuroradiological manifestations of drug use: description of clinical cases

**DOI:** 10.1192/j.eurpsy.2023.1598

**Published:** 2023-07-19

**Authors:** I. Faria, T. Silva

**Affiliations:** Psychiatry, Coimbra Hospital and University Centre, Coimbra, Portugal

## Abstract

**Introduction:**

Drug use and misuse continue to create public health challenges in the world, leading to overdose deaths, infections, and other chronic health conditions. Illegal addictive drugs can lead to functional or structural impairment of the central nervous system (CNS). Because clinical findings alone are often nonspecific, and some patients are unlikely to admit substance abuse, the neuroimaging may play an important role in establishing the diagnosis and initiating treatment.

**Objectives:**

We aim to provide an overview of the structural imaging findings on computed tomography (CT), magnetic resonance (MR) imaging related to chronic and acute abuse of commonly addictive substances, including cannabis, alcohol, cocaine, and opioids.

**Methods:**

Non systematic review of the literature on the subject and description of three clinical cases.

**Results:**

Pathomecanisms of drugs misuse include excitotoxicity, which may lead to an acute or subacute leukoencephalopathy, and vascular complications, including vasoconstriction, vasculitis, or hypertension, which may lead to intracranial hemorrhage or ischemia. Alcohol abuse may lead to Wernicke-Korsakoff syndrome, revealing in MR bilateral symmetrical hyperintense signals on T2-weighted; Marchiafava-Bignami disease (MBD) is a very rare condition which may present hypodense lesions in the corpus callosum; and alcoholic cerebellar degeneration is a common type of acquired cerebellar ataxia and may present cerebellar volume loss localized to the anterior superior vermis. One of our clinical cases is a female, 39 years, and present cocaine induced multifocal leukoencephalopathy, associated with inflammatory/immune mediated mechanism. Other clinical case (female, 24 years) demonstrate spongiform leukoencephalotpathy from “chasing” heroin, with a characteristic presentation.

**Conclusions:**

The main pathomechanisms related to the abuse of drugs are ischemia, hemorrhage, and leukoencephalopathy related to excitotoxicity of the drug or its derivatives. Clinical findings are nonspecific, highlighting the need to recognize these complications at both CT and MR imaging. Therefore, diagnostic imaging modalities can play a pivotal role in the recognition and timely management of drug-related complications in the CNS.

**Disclosure of Interest:**

None Declared

